# Classification of three-level hybrid surgery for the treatment of cervical degenerative disc disease: a retrospective study of 108 patients

**DOI:** 10.1186/s12893-022-01627-7

**Published:** 2022-05-14

**Authors:** Kangkang Huang, Han Wang, Hao Liu, Yang Meng, Chen Ding, Beiyu Wang, Tingkui Wu, Ying Hong

**Affiliations:** 1 Department of Orthopedics, West China Hospital, Sichuan University, 37# Guoxue Lane, Chengdu, 610041 Sichuan Province China; 2 West China School of Nursing, Sichuan University, Chengdu, 610041 Sichuan Province China; 3 Department of Operation Room, West China Hospital, Sichuan University, Chengdu, 610041 Sichuan Province China

**Keywords:** Cervical degenerative disc disease, Three-level, Hybrid surgery, Constructs, Classification

## Abstract

**Introduction:**

According to the different numbers and locations of cervical disc arthroplasty (CDA) and anterior cervical discectomy and fusion (ACDF), three-level hybrid surgery (HS) has many constructs. The purpose of the present study was to introduce a classification system for three-level HS and compare the two types with each other and with ACDF.

**Methods:**

A retrospective study was conducted involving patients with three-level cervical degenerative disc disease (CDDD) who underwent ACDF or HS in our hospital between June 2012 and May 2019. According to the different numbers and locations of ACDFs and CDAs, we classified the three-level HS into two types (type I: one-level CDA and two-level ACDF, and type II: two-level CDA and one-level ACDF). The differences of clinical and radiological outcomes were compared with each other and with three-level ACDF.

**Results:**

A total of 108 patients were analyzed. The Neck Disability Index (NDI) of the ACDF group at 3 months postoperatively was significantly higher than that in the type I and type II groups (p < 0.05). The cervical lordosis was significantly lower in the ACDF group than that in the type I and II groups at 3 days, 6, 12 months postoperatively and the final follow-up (p < 0.05). The range of motion (ROM) of the total cervical spine decreased significantly in all three groups at 3, 6, and 12 months postoperatively and at the final follow-up (p < 0.05). The ACDF group was observed with the most severe loss of ROM of the total cervical spine, followed by the type I group. The type II group could preserve the most ROM of the total cervical spine. The ROM of adjacent segments increased most in the ACDF group, followed by the type I group.

**Conclusions:**

Compared with ACDF, three-level HS may yield a faster recovery rate and superior radiological outcomes, such as a superiority in maintaining the cervical curvature and ROM of the total cervical spine and a smaller increase in the ROM of adjacent segments. The advantages were most remarkable in the type II group.

## Introduction

Due to the development of individual therapy and precision medicine, anterior cervical discectomy and fusion (ACDF), which is considered a traditional surgery, has been partly replaced by cervical disc arthroplasty (CDA) [1–4] Hybrid surgery (HS), a combination of ACDF and CDA, has also been used for the treatment of multilevel cervical degenerative disc disease (CDDD). Many studies have indicated that HS is a safe, effective and reliable surgical procedure [5–9]. For three-level HS, a systematic review and meta-analysis of four three-level HS studies showed that HS is a novel surgical approach to treat multilevel CDDD and that compared with ACDF, it is associated with better preservation of range of motion (ROM), a longer operative time, less intraoperative blood loss and comparable if not superior clinical outcomes [10].

Unlike two-level HS, according to the different numbers and locations of CDAs and ACDFs, three-level HS has many constructs. Some surgeons have demonstrated the differences among different constructs [11–14]. However, most of the studies are finite element analysis. Moreover, in previous studies, these constructs were defined with various designations. To facilitate academic communication, we classified the different constructs of three-level HS. The purpose of the present study was to introduce a classification system for three-level HS and compare the two types with each other and with ACDF.

## Methods

### Population information

**Fig. 1:**
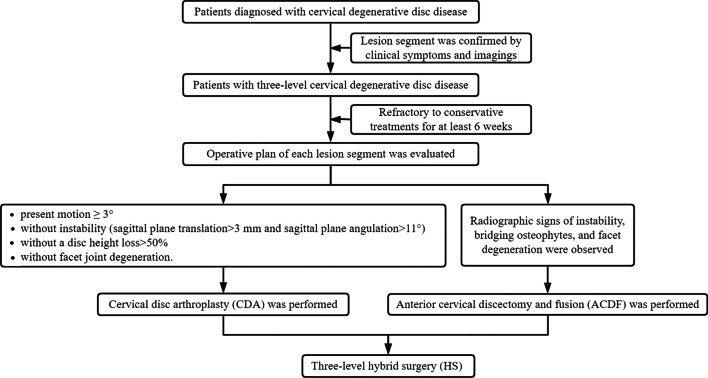
Decision-making algorithm of surgical indication and surgical methods

A retrospective study was conducted involving patients with three-level CDDD who underwent ACDF or HS in our hospital between June 2012 and May 2019. The inclusion criteria consisted of (1) a diagnosis of cervical myelopathy and radiculopathy; (2) refractory to conservative treatments for at least 6 weeks; (3) lesion segment was confirmed by clinical symptoms and imagings (computed tomography, magnetic resonance imaging, and radiography); (4) surgery on three levels between C3 and C7; and (5) surgery performed by CDA with a Prestige-LP system (Medtronic Sofamor Danek, Memphis, TN, USA) and/or ACDF with a Zero-P implant system (Synthes, Oberdorf, Switzerland) [15]. The exclusion criteria consisted of (1) previous surgery at cervical spine; (2) existence of cervical stenosis, osteoporosis, tumor, and infection. The indications of CDA at lesion segment was according with previous studies, which were without instability (sagittal plane translation > 3 mm and sagittal plane angulation > 11°), without an absence of motion < 3°, without a disc height loss > 50%, and without facet joint degeneration [6]. If instability, bridging osteophytes, and facet degeneration were observed at the radiological images, ACDF was performed (Fig. 1) [15]. Ethical approval was given by the medical ethics committee of our hospital (No. 2019-567).

### Classification

**Fig. 2:**
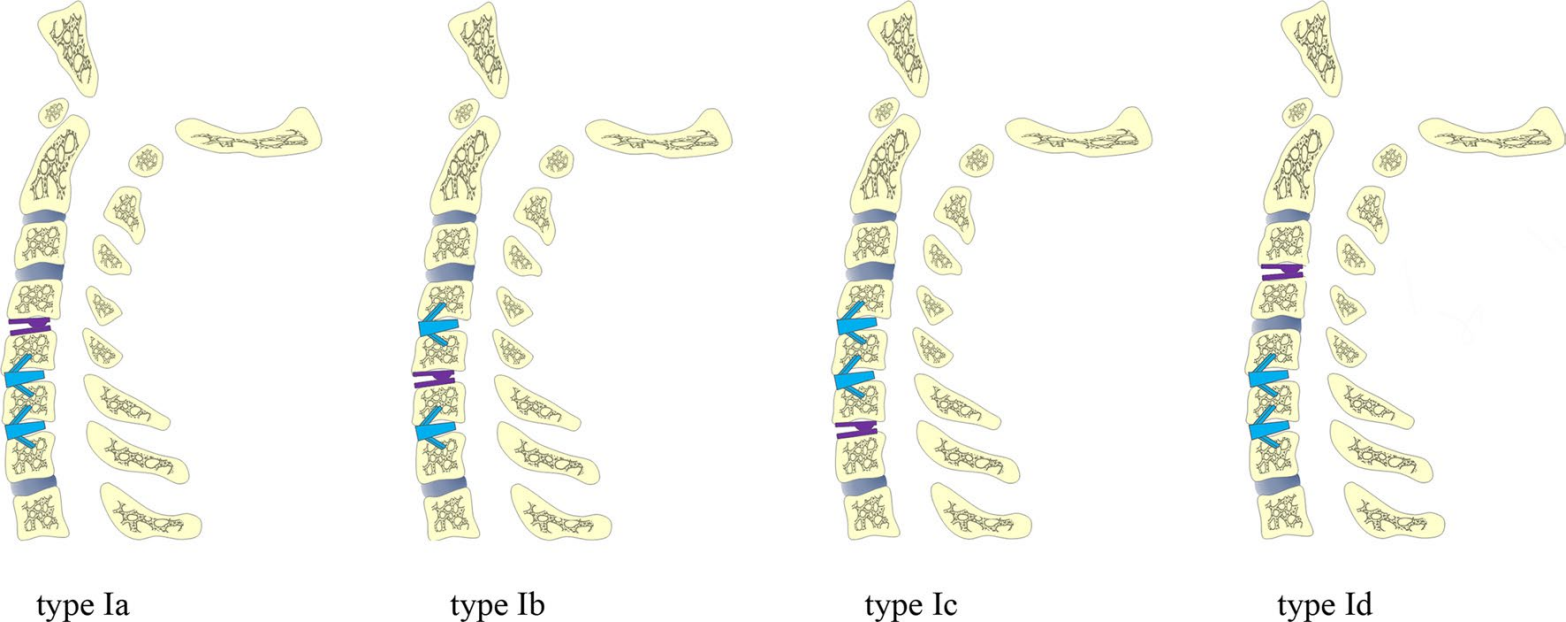
Sketches of type I three-level hybrid surgery (HS). The subtypes are classified according to the different locations of cervical disc arthroplasty (CDA) and anterior cervical discectomy and fusion (ACDF)

**Fig. 3:**
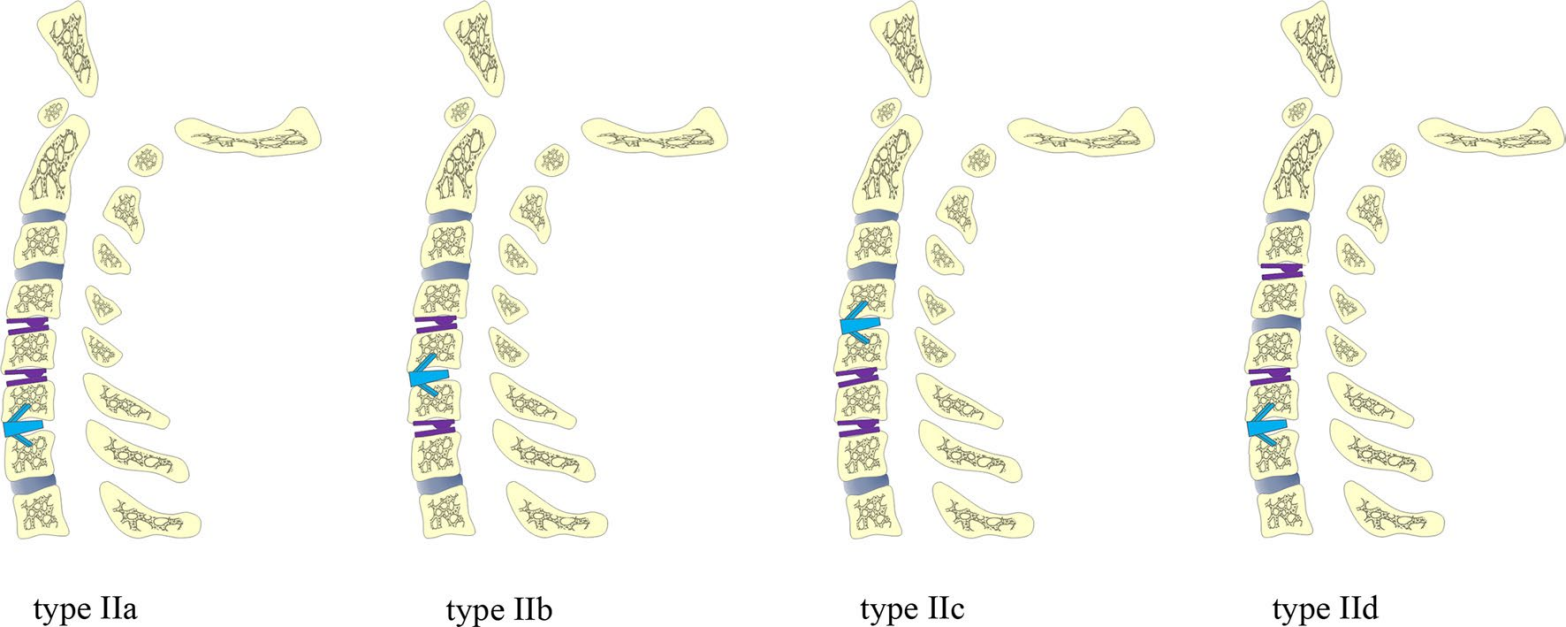
Sketches of type II three-level hybrid surgery (HS). The subtypes are classified according to the different locations of cervical disc arthroplasty (CDA) and anterior cervical discectomy and fusion (ACDF)

According to the different numbers and locations of ACDFs and CDAs, we classified three-level HS into two types with eight subtypes (Table 1; Figs. 2, 3). Based on the classification, the patients were divided into three groups, type I group, type II group and ACDF group.

**Table 1:** the classification of three-level HS

Classification	Description
Type I	One-level CDA and two-level ACDF
Type Ia	Contiguous three-level HS. CDA was performed at the superior lesion segment, and ACDF was performed at the intermediate and inferior lesion segments
Type Ib	Contiguous three-level HS. CDA was performed at the intermediate lesion segment, and ACDF was performed at the superior and inferior lesion segments
Type Ic	Contiguous three-level HS. CDA was performed at the inferior lesion segment, and ACDF was performed at the superior and intermediate lesion segments
Type Id	Noncontiguous three-level HS
Type II	Two-level CDA and one-level ACDF
Type IIa	Contiguous three-level HS. CDA was performed at the superior and intermediate lesion segments, and ACDF was performed at the inferior lesion segment
Type IIb	Contiguous three-level HS. CDA was performed at the superior and inferior lesion segments, and ACDF was performed at the intermediate lesion segment
Type IIc	Contiguous three-level HS. CDA was performed at the intermediate and inferior lesion segments, and ACDF was performed at the superior lesion segment
Type IId	Noncontiguous three-level HS

*HS* hybrid surgery, *CDA* cervical disc arthroplasty, *ACDF* anterior cervical discectomy and fusion

### Surgical technique

All operations were performed by the same senior spine surgeon (HL). The procedure was carried out as described previously [15]. Briefly, the operation was performed through a standard right-sided anterior cervical approach. After complete discectomy of all target levels, the end plates of CDA level were well prepared, a properly sized Prestige-LP disc was inserted along with channels in the end plates. Then, an appropriate size of Zero-P implant system packed with β-tricalcium phosphate was inserted into the ACDF level. C-arm fluoroscopy was performed to verify the proper placement of the implants. Finally, the incision was closed after the insertion of a drain.

### Data collection

The data were collected preoperatively and at 3 days, 3 months, 6 months, and 12 months postoperatively and at the final follow-up. Perioperative parameters, including the operative time and blood loss, were collected.

### Clinical evaluation

The Japanese Orthopedic Association (JOA) scores, Neck Disability Index (NDI), and visual analog scale (VAS) scores of the neck and arm were used for the evaluation of clinical outcomes. The JOA scores were used to evaluate functional recovery of the nerve, the NDI was used to evaluate neck function, and the VAS scores were used to evaluate the pain intensity of the neck and arm.

### Radiological evaluation

**Fig. 4:**
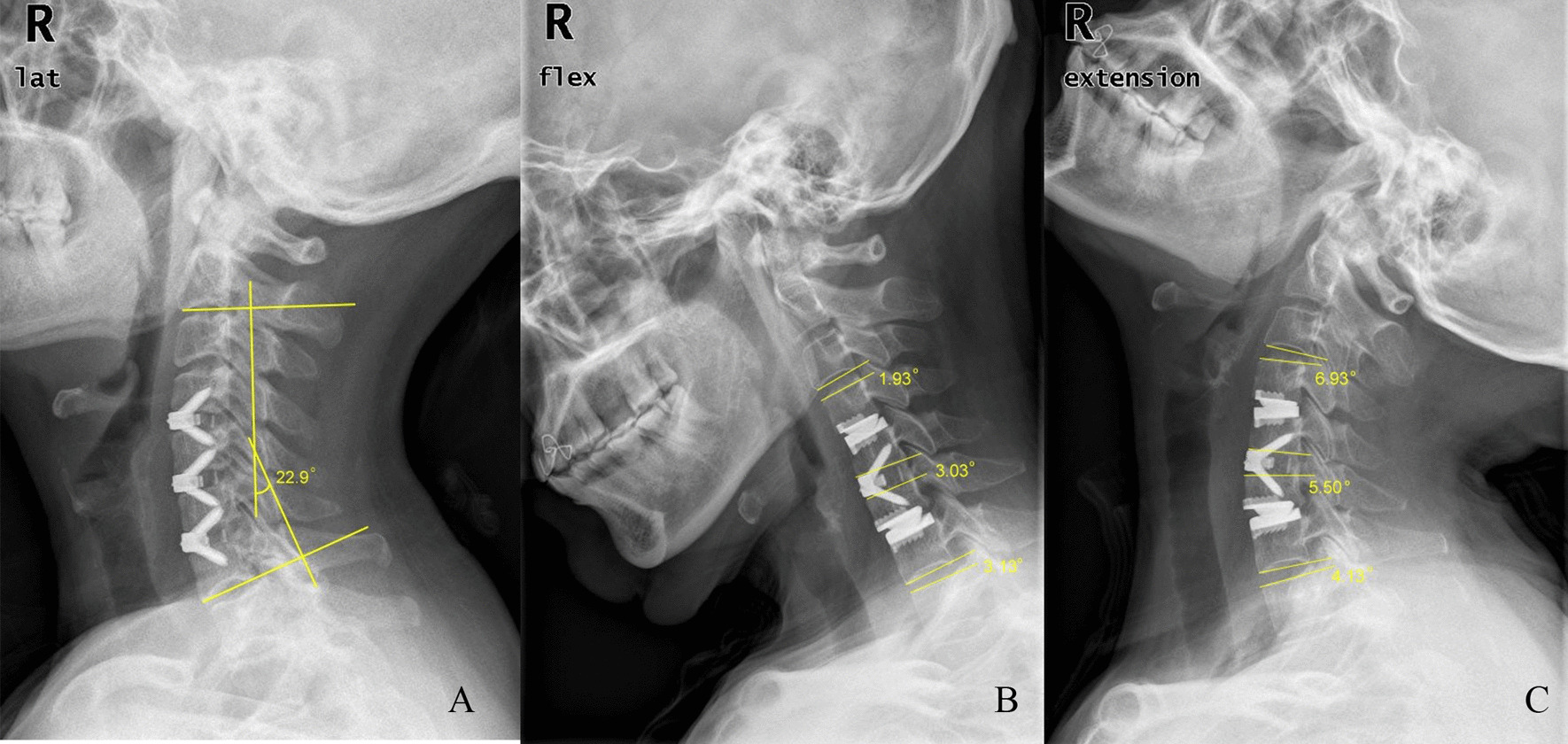
The Cobb method for the measurement of cervical lordosis (CL) (**A**), and measurement of range of motion (ROM) at the fusion and adjacent segments (**B**, **C**)

Radiological evaluations were conducted via lateral radiographs for flexion, extension, and neutral positions. Cervical lordosis (CL), ROM of the total cervical spine, and ROM of the adjacent segments were measured using the Cobb method (Fig. 4) [16]. Fusion was considered according to the following accepted criteria [17]: (1) less than 2º of segmental movement on lateral flexion/extension views, (2) absence of a radiolucent gap between the graft and endplates, and (3) presence of continuous bridging bony trabeculae at the graft endplate interface.

The incidences of complications, including dysphagia, hematoma, screw loosening, device migration, or adjacent segment disease (ASD) were also recorded. ASD was defined as new disease affecting the level superior and/or inferior to the operated levels that required surgery [18].

The clinical evaluation was performed in a blinded fashion by two spine surgeons (HW and TKW) who was absent in the surgical procedures. The radiological evaluation was performed by two independent spine surgeons (KKH and BYW), and the mean values were used for statistical analysis. The radiological evaluation was not blinded because the fixation device allowed the assessors to identify the group to which the patients belonged.

### Statistical analysis

All statistical analyses were performed using SPSS (version 19.0, SPSS, Chicago, IL, USA). The findings are presented as the mean values ± standard deviation (SD) or counts, as indicated. ANOVA and Student–Newman–Keuls (SNK) tests were applied to compare the clinical and radiographic effects as qualitative data among the three groups. A paired t-test was used to assess changes between preoperative and postoperative parameters. A Chi-square test or Fisher’s exact test was used to analyze categorical data. Statistical significance was defined as p < 0.05.

## Results

### Demographic and surgical data

According to the inclusion and exclusion criteria, a total of 108 patients were included in the analysis, with 50 patients in the type I group, 34 in the type II group and 24 patients in the ACDF group. There were no significant differences in the sex ratio, body mass index (BMI), the distribution of the surgical levels, average blood loss, or the average follow-up time among the three groups. The average age of the ACDF group was significantly older than those of the type I and type II groups (p < 0.05). The operation time in the type II group was 178.82 ± 23.06 min, which was significantly longer than that in the ACDF and type I groups (p < 0.05). However, no significant difference was observed between the ACDF and type I groups. The detailed information is shown in Table 2.

**Table 2:** Summary of the patient demographic data

	Type I	Type II	ACDF	p value	p value		
					I vs. II	I vs. ACDF	II vs. ACDF
N	50	34	24				
Gender, n^a^							
Male Female	26 24	17 17	13 11	0.952	0.857	0.861	0.755
Age, year^b^	51.83 ± 6.86	48.94 ± 8.93	56.67 ± 7.93	**0.001**	0.098	**0.013**	**0.000**
BMI^b^	24.27 ± 2.92	23.29 ± 2.64	23.61 ± 3.29	0.318	0.142	0.373	0.681
Levels, n^a^							
C3–6	12	12	10	0.569	0.471	0.297	0.839
C4–7	35	21	13				
Skip	3	1	1				
Operation time, min^b^	165.65 ± 23.25	178.82 ± 23.06	154.58 ± 25.70	**0.001**	**0.016**	0.067	**0.000**
Blood loss, ml^b^	67.83 ± 28.04	75.00 ± 22.05	61.67 ± 23.53	0.138	0.211	0.334	0.050
FU, months^b^	29.13 ± 14.40	34.03 ± 19.74	29.13 ± 14.19	0.359	0.187	0.999	0.262

*ACDF* anterior cervical discectomy and fusion, *BMI* body mass index, *FU* follow-up

^a^Chi-square test for the three groups

^b^ANOVA test for the three groups and SNK test for each two groups

### Clinical outcomes

The JOA scores were increased in all three groups at 3 months after surgery (p < 0.001) and continued to improve during the follow-up period. However, no significant differences were found among the three groups at any follow-up point. Analogously, the VAS scores of the neck and arm were decreased in all three groups at 3 months after surgery (p < 0.001), and continued to improve during the follow-up period. However, no significant differences were found among the three groups at any follow-up point. The NDI scores also decreased in all three groups postoperatively (p < 0.001). However, the NDI score of the ACDF group at 3 months postoperatively was 17.79 ± 2.48, which was significantly higher than those of the type I and type II groups (p < 0.05). No significant differences were found among the three groups at the other follow-up points. The detailed information of each scale is shown in Table 3.

**Table 3:** Clinical outcomes

	Type I	Type II	ACDF	p value*	p value†		
					I vs. II	I vs. ACDF	II vs. ACDF
VAS score of neck							
Pre-op	5.04 ± 1.32	4.59 ± 1.35	5.13 ± 1.23	0.209	0.127	0.805	0.127
Po-3 m	2.33 ± 0.63	2.62 ± 1.02	2.5 ± 0.72	0.265	0.109	0.388	0.581
Po-6 m	1.98 ± 0.61	2.06 ± 0.65	1.83 ± 0.48	0.370	0.553	0.339	0.161
Po-12 m	1.5 ± 0.84	1.76 ± 0.89	1.42 ± 0.72	0.226	0.161	0.691	0.119
The final	0.87 ± 0.78	1 ± 0.7	0.83 ± 0.64	0.627	0.426	0.842	0.388
VAS score of arms							
Pre-op	4.15 ± 1.61	4.41 ± 1.86	4.83 ± 1.09	0.242	0.474	0.093	0.324
Po-3 m	1.87 ± 0.93	1.88 ± 0.81	2 ± 0.78	0.822	0.948	0.548	0.609
Po-6 m	1.26 ± 0.99	1.59 ± 0.92	1.29 ± 0.75	0.262	0.120	0.895	0.231
Po-12 m	1.13 ± 0.96	1.29 ± 0.84	1.08 ± 0.72	0.600	0.406	0.830	0.365
The final	0.87 ± 1.13	0.79 ± 0.64	0.54 ± 0.59	0.334	0.706	0.143	0.286
JOA score							
Pre-op	10.17 ± 1.34	10.06 ± 1.13	9.75 ± 0.74	0.348	0.661	0.148	0.319
Po-3 m	14.24 ± 1.14	13.88 ± 1.09	14.04 ± 0.95	0.346	0.149	0.471	0.583
Po-6 m	14.83 ± 1.27	14.76 ± 0.99	15.04 ± 0.95	0.631	0.808	0.445	0.354
Po-12 m	15.39 ± 1.32	15.12 ± 0.98	15.5 ± 0.88	0.393	0.286	0.703	0.207
The final	16.2 ± 1.28	16.24 ± 0.82	16.42 ± 0.78	0.692	0.867	0.401	0.514
NDI							
Pre-op	29.67 ± 4.12	28.12 ± 4.37	29.92 ± 4.02	0.173	0.103	0.818	0.110
Po-3 m	15.54 ± 3.66	15.65 ± 3.87	17.79 ± 2.48	**0.029**	0.896	**0.012**	**0.024**
Po-6 m	11.65 ± 3.99	11.59 ± 3.91	12.54 ± 2.5	0.564	0.939	0.339	0.333
Po-12 m	8.3 ± 4.13	9.56 ± 4.26	9.92 ± 2.81	0.187	0.159	0.105	0.732
The final	5 ± 5.91	5.47 ± 3.37	6.29 ± 2.53	0.532	0.649	0.262	0.500

*ACDF* anterior cervical discectomy and fusion, *JOA* Japanese Orthopedic Association, *NDI* neck disability index, *VAS* visual analog scale

^*^ANOVA test

^†^SNK test

### Radiological outcomes

#### Cervical lordosis and ROM of the total cervical spine

**Fig. 5:**
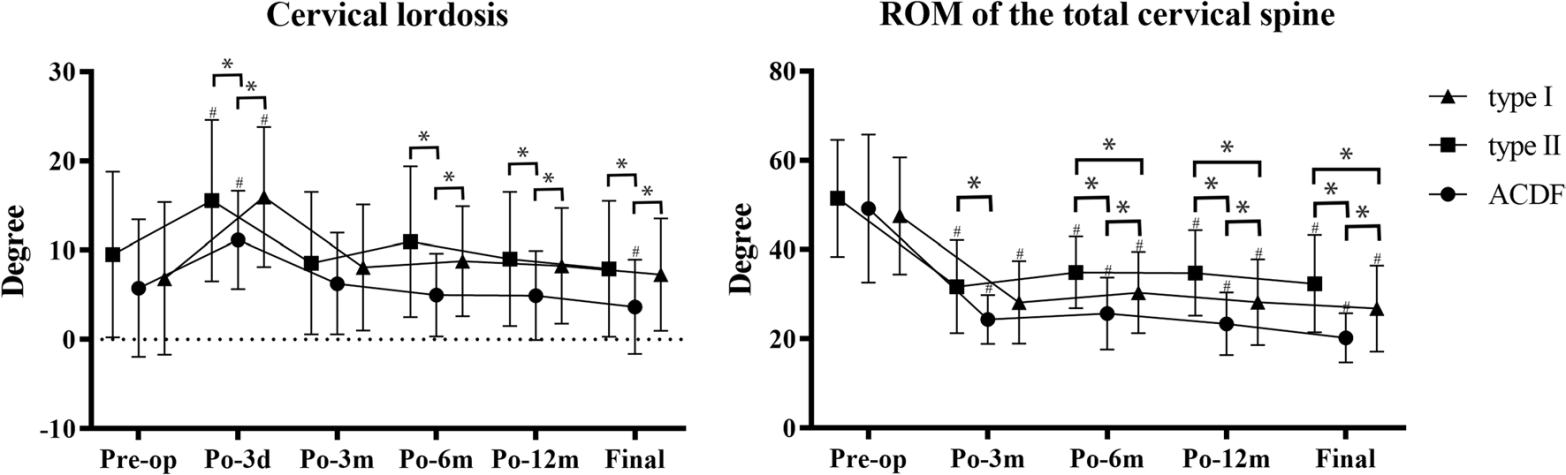
The cervical lordosis (CL) and range of motion (ROM) of the total cervical spine. (#p < 0.05 compared with preoperative value, *p < 0.05 between two groups). The CL was significantly lower in the ACDF group than that in the type I and II groups at 3 days, 6, and 12 months postoperatively and at the final follow-up (p < 0.05). The ROM of the total cervical spine decreased significantly in all three groups at 3, 6, and 12 months postoperatively and at the final follow-up (p < 0.05). It was decreased mostly in ACDF group, followed by the type I group (p < 0.05). The type II group could preserve the most ROM of the total cervical spine (p < 0.05)

For the type I group, the CL significantly increased from 6.83 ± 8.54° preoperatively to 15.95 ± 7.83° postoperatively (p < 0.05). However, it decreased at 3 months postoperatively to a degree that was not significantly different from that preoperatively or at the other follow-up points. For the type II and ACDF groups, a similar trend was observed. However, for the ACDF group, the CL was 3.66 ± 8.54° at the final follow-up, which was significantly lower than that preoperatively (p < 0.05). Moreover, the CL was significantly lower in the ACDF group than that in the type I and II groups at 3 days, 6, and 12 months postoperatively and at the final follow-up (p < 0.05). The ROM of the total cervical spine decreased significantly in all three groups at 3, 6, and 12 months postoperatively and at the final follow-up (p < 0.05). The ROM of the total cervical spine in the type I group was 30.39 ± 9.11° at 6 months postoperatively, 28.21 ± 9.59° at 12 months postoperatively, and 26.82 ± 9.64° at the final follow-up. For the type II group, the corresponding values were 34.91 ± 8.01°, 34.76 ± 9.53° and 32.38 ± 10.88°, respectively. For the ACDF group, the corresponding values were 25.67 ± 8.07°, 23.39 ± 7.01° and 20.25 ± 5.52°, respectively. Significant differences were found between each two groups (p < 0.05). The changes over the follow-up period are shown in Fig. 5.

#### ROM of the adjacent segments

**Fig. 6:**
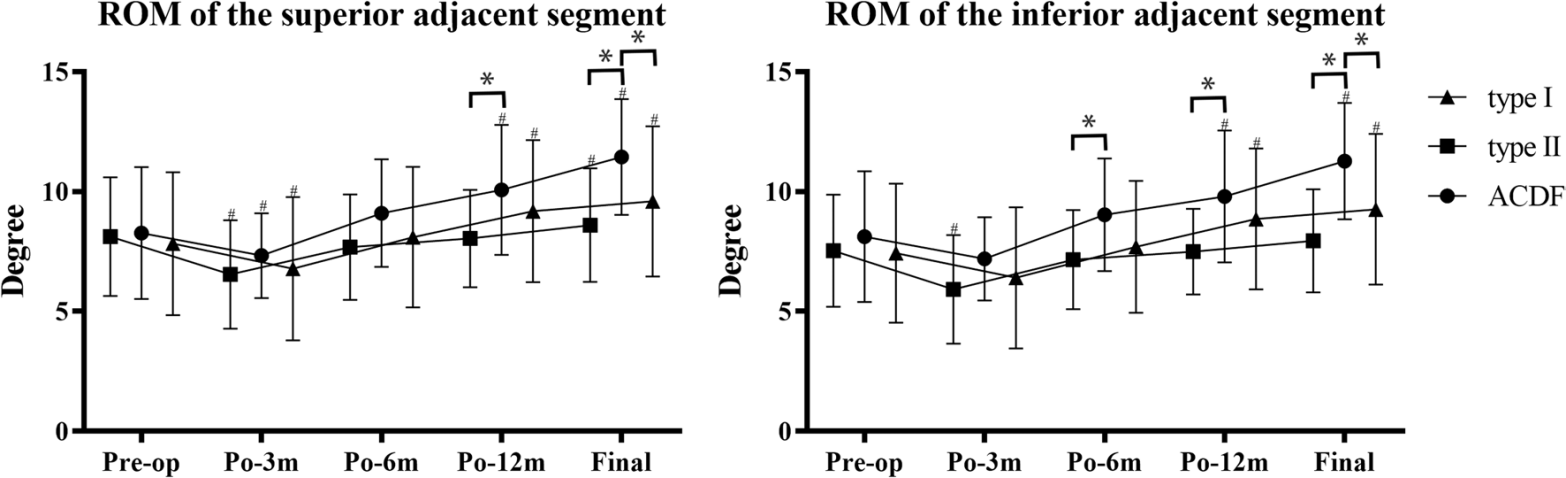
The range of motion (ROM) of adjacent segments. (#p < 0.05 compared with preoperative value, *p < .05 between two groups). The ROM of the superior adjacent segment increased significantly in all three groups at the final follow-up compared with the preoperative data (p < 0.05). It was significantly higher in the ACDF group than those in the type I and type II groups at the final follow-up (p < 0.05). For the ROM of the inferior adjacent segment, a significant increase was observed in the type I and ACDF groups (p < 0.05). It was also significantly higher in the ACDF group at the final follow-up than those in the type I and type II groups (p < 0.05)

In the analysis of ROM of the adjacent segments, the patients who underwent with skip-level procedures were excluded (3 patients in the type I group, 1 patient in the type II group, and 1 patient in the ACDF group). In addition, not all of the data of the inferior adjacent segments were recorded because the shoulders occluded the measurement in some patients. Six patients in the type I group, 3 patients in the type II group, and 2 patients in the ACDF group were excluded. For ROM of the superior adjacent segment, a significant increase was observed in all three groups at the final follow-up compared with the preoperative data (p < 0.05). At the final follow-up, the ROM of the superior adjacent segment in the ACDF group was 11.44 ± 2.41°, which was significantly higher than those in the type I and type II groups (p < 0.05). For the ROM of the inferior adjacent segment, a significant increase was also observed in the type I and ACDF groups (p < 0.05). However, the ROM of the inferior adjacent segment in the type II group at the final follow-up was 7.94 ± 2.15°, which was not significantly different from that preoperatively. Similar to the ROM of the superior adjacent segment, the ROM of the inferior adjacent segment was significantly higher in the ACDF group at the final follow-up than those in the type I and type II groups (p < 0.05). The changes over the follow-up period are shown in Fig. 6.

#### Fusion rate

At the final follow-up, solid fusion was observed in 46 patients (92%) in the type I group, 31 patients (91.2%) in the type II group, and 22 patients (91.7%) in the ACDF group, without significant difference between each two groups.

#### Complications

**Fig. 7:**
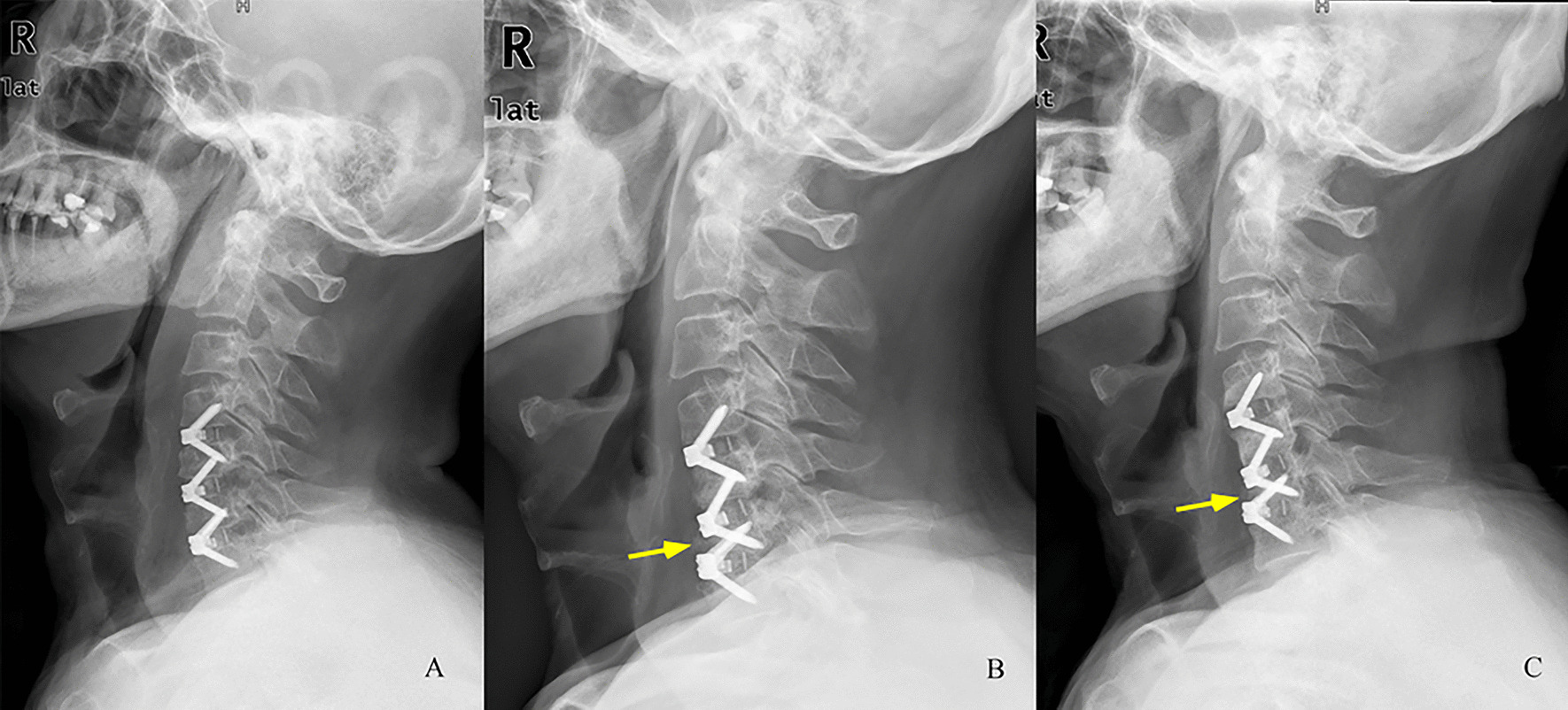
A 68-year-old man underwent three-level anterior cervical discectomy and fusion (ACDF) with Zero-p implant system. The lateral X-ray 3 days after surgery showed the prostheses were implanted well (**A**). However, the severe collapsibility phenomenon could be observed at C6 vertebral body 3 months postoperatively (**B**). The lateral X-ray 18 months postoperatively showed the collapsibility didn’t become aggravated after the solid fusion achieved (**C**)

Dysphagia was reported in 13 patients in the type I group, 10 patients in the type II group, and 7 patients in the ACDF group after surgery, and all patients recovered within 6 months. There were no significant differences between each two groups. Two patients in type the I group underwent revision surgery due to ASD during the follow-up period. Two patients in the ACDF group exhibited a significant collapsibility of the surgical vertebral bodies during the follow-up period (Fig. 7). The collapsibility was interrupted after solid fusion achieved. The patients were asymptomatic, so no revision surgeries were performed. No cases of hematoma, screw loosening, or device migration was observed after surgery or during the follow-up period.

## Discussion

The surgical plan for multilevel CDDD is still controversial. A meta-analysis and systematic review showed that the anterior approach group had a significantly higher JOA score and neurological recovery rate than did the posterior approach group [19]. However, the ROM of the cervical spine was significantly lower in the patients treated with the anterior approach [20]. In some patients with multilevel CDDD, the diseased levels may not show the same type or degree of degeneration. Thus, HS was performed, achieving a satisfactory clinical outcome and preserving the ROM of the cervical spine. We have previously compared HS with posterior cervical laminoplasty. The results showed that the cervical curvature was significantly higher in the HS group but that the ROM of the cervical spine was not significantly different. Although the early complication rate was higher in the HS group, the late complication rate was lower [21].

For three-level CDDD, the safety and efficacy of HS were compared with those of ACDF in many studies [22–25]. The clinical outcomes were satisfactory, and the ROM of the cervical spine seemed to be preserved. In addition, the influence on adjacent segments may be lower with HS. Although the follow-up time of three-level HS was relatively short, many surgeons accept HS as an alternative surgical procedure. More studies are being conducted to improve the efficacy of HS.

According to the different numbers and locations of CDAs and ACDFs, three-level HS has several constructs. In previous studies, the biomechanical properties of different constructs were analyzed through finite element analysis. Li et al. [11] compared the biomechanical properties of three-level HS concerning one-level ACDF and two-level CDA. They found that the CDA-CDA-ACDF construct may lead to more compensation in terms of motion and facet stress. The biomechanical differences of three-level HS concerning one-level CDA and two-level ACDF was employed in Xie et al. [12] study. The results showed that the ACDF-CDA-ACDF construct resulted in a better theoretical outcome, especially in preserving the maximum total ROM. In Wong et al. study, all six constructs were compared both among intergroup and with three-level ACDF and three-level CDA [13]. In conclusion, the authors recommended the ACDF-CDA-CDA construct and three-level CDA for patients with C3–C6 disc degeneration without predisposing C2-3 conditions, and the ACDF-ACDF-CDA construct could be a good alternative with a lower medical cost. Xu et al. [14] performed a clinical study that took different numbers of CDAs into consideration and compared the clinical outcomes and sagittal alignment among three-level ACDF, one-level ACDF combined with two-level CDA and one-level CDA combined with two-level ACDF. In the authors’ opinions, it was not necessary to performed CDA for three-level cervical spondylotic myelopathy (CSM). In these previous studies, different definitions of three-level HS constructs were used. In the future, different constructs of three-level HS will be explored in detail in many studies involving many surgeons. Thus, we think it is necessary to define the different constructs of three-level HS for better academic communication and consistency among the studies. In the present study, according to the different numbers and locations of CDAs and ACDFs, we classified three-level HS into two types and eight subtypes. The differences among type I, type II and three-level ACDF were studied.

As a result, we found that the average age of the patients in the ACDF group was significantly higher than those in the type I and type II groups. The reason may be that CDA was performed in patients between 18 and 60 years old. Patients older than 60 years old usually underwent ACDF. In addition, the average operation time was significantly lower in the ACDF group because CDA required more steps. All the clinical outcomes improved after surgery, showing no significant differences among groups, except for NDI at 3 months after surgery, which means that HS may yield a faster recovery rate.

The preponderance of HS was remarkable in our study. CL improved after surgery in all three groups, but the loss of CL could be observed during the follow-up. This finding may be due to the insufficiency of zero-profile implants in maintaining the curvature [26]. However, the degree of CL in the ACDF group significantly decreased at the final follow-up, and was lower than those in the type I and type II groups. This finding indicated that the Prestige LP implant may be superior in maintaining the cervical curvature than the Zero-P implant. The ROM of the total cervical spine was significantly higher in the HS groups than the ACDF group. Moreover, one more CDA in three-level HS could significantly retain more ROM of the total cervical spine. For the ROM of adjacent segments, the results showed that HS may have a smaller influence than ACDF. These results indicated that HS may yield superior radiological outcomes for the treatment of three-level CDDD. The superiority may have a positive impact on the long-term effect. In the ACDF group, the severe collapsibility phenomenon of surgical vertebral bodies was observed in two patients. We think this phenomenon may be related to the biomechanical consequences to the vertebral body located between the two adjacent fusion levels. A similar phenomenon was also reported in Mattei et al. study [27]. The biomechanical characteristics of the vertebral body located between the two adjacent fusion levels will be explored in the future.

The present study had several limitations. First, due to the retrospective design, there may be bias. In addition, the different average ages of the patients among groups may have also biased the results. Second, the sample size was relatively small, and the follow-up was relatively short. Third, only the Zero-P and Prestige-LP system were included in the study. In the future, prospective, multicenter, large-scale studies concerning different prostheses should be performed to confirm the results.

## Conclusions

Compared with ACDF, three-level HS may yield a faster recovery rate and superior radiological outcomes, such as a superiority in maintaining the cervical curvature and ROM of the total cervical spine and a smaller increase in the ROM of adjacent segments. The advantages were most remarkable in the type II group.

## Data Availability

Summarized data have been presented in this manuscript. The raw data for this study are located and protected at West China Hospital of Sichuan University. Sharing of the raw data is not suggested, because a secondary analysis is planned.

## Abbreviations

ACDF: Anterior cervical discectomy and fusion
CDA: Cervical disc arthroplasty
HS: Hybrid surgery
CDDD: Cervical degenerative disc disease
ROM: Range of motion
JOA: Japanese Orthopedic Association
NDI: Neck Disability Index
VAS: Visual analog scale
CL: Cervical lordosis
ASD: Adjacent segment disease
SD: Standard deviation
SNK: Student–Newman–Keuls
BMI: Body mass index
